# Efficacy, durability, and safety of faricimab up to every 16 weeks in patients with neovascular age-related macular degeneration: 2-year results from the Japan subgroup of the phase III TENAYA trial

**DOI:** 10.1007/s00417-024-06377-1

**Published:** 2024-03-14

**Authors:** Hideki Koizumi, Fumi Gomi, Akitaka Tsujikawa, Shigeru Honda, Ryusaburo Mori, Haruka Ochi, Keisuke Iwasaki, Annabelle Ayame Okada, for the TENAYA and LUCERNE Investigators

**Affiliations:** 1 Department of Ophthalmology, Graduate School of Medicine, University of the Ryukyus, Okinawa, Japan; 2 Department of Ophthalmology, Hyogo Medical University, Hyogo, Japan; 3 Department of Ophthalmology and Visual Sciences, Kyoto University Graduate School of Medicine, Kyoto, Japan; 4 Department of Ophthalmology and Visual Sciences, Graduate School of Medicine, Osaka Metropolitan University, Osaka, Japan; 5 Department of Ophthalmology, Nihon University School of Medicine, Tokyo, Japan; 6 Chugai Pharmaceutical Co., Ltd., Tokyo, Japan; 7 Department of Ophthalmology, Kyorin University School of Medicine, Tokyo, Japan

**Keywords:** Angiopoietin-2, Anti-VEGF therapy, Choroidal neovascularisation, Faricimab, Neovascular age-related macular degeneration

## Abstract

**Purpose:**

To evaluate 2-year efficacy, durability, and safety of faricimab in the TENAYA Japan subgroup and pooled global TENAYA/LUCERNE cohort of patients with neovascular age-related macular degeneration (nAMD).

**Methods:**

Subgroup analysis of TENAYA/LUCERNE (NCT03823287/NCT03823300): phase III, multicentre, randomised, active comparator–controlled, double-masked, non-inferiority trials. Treatment-naïve patients aged ≥ 50 years with nAMD were randomised (1:1) to intravitreal faricimab (6.0 mg up to every 16 weeks [Q16W] after 4 initial Q4W doses) or aflibercept (2.0 mg Q8W after 3 initial Q4W doses). Outcomes were assessed through year 2 for the TENAYA Japan subgroup (*N* = 133) and global pooled TENAYA/LUCERNE cohort (*N* = 1329).

**Results:**

Vision and anatomic improvements achieved with faricimab at year 1 were maintained over 2 years and were generally comparable between the TENAYA Japan subgroup and pooled TENAYA/LUCERNE cohort. Adjusted mean best-corrected visual acuity (BCVA) change from baseline at year 2 for the TENAYA Japan subgroup and global pooled TENAYA/LUCERNE cohort was +7.1 (3.7–10.5) and +4.4 (3.2–5.5) letters in the faricimab arm, respectively, and +5.2 (1.9–8.6) and +4.3 (3.1–5.4) letters in the aflibercept arm, respectively. At week 112, the proportion of faricimab-treated patients on Q16W dosing was 61.0% and 63.1% in the TENAYA Japan subgroup and pooled TENAYA/LUCERNE cohort. Faricimab was well tolerated through year 2.

**Conclusion:**

Year 2 TENAYA Japan subgroup findings for faricimab were generally consistent with the pooled global TENAYA/LUCERNE results in patients with nAMD. Vision and anatomical benefits with faricimab were similar to those with aflibercept but with fewer injections.

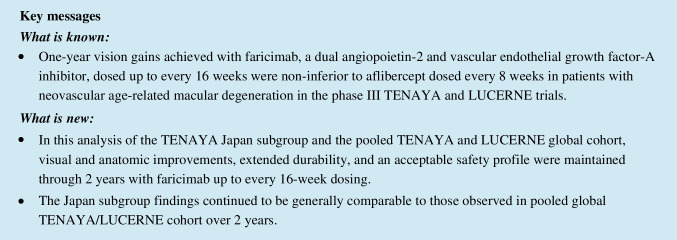

**Supplementary Information:**

The online version contains supplementary material available at 10.1007/s00417-024-06377-1.

## Introduction

Age-related macular degeneration (AMD) is a leading cause of blindness globally in adults aged 60 years and older [1]. In Japan, nAMD has a high prevalence in older populations (1% of those aged 70–74 years vs 0.3% of those aged 50–59 years) [2]. Between 2005 and 2013, the proportion of patients with nAMD in Japan increased by approximately three-fold [3]. As nAMD commonly causes vision impairment and legal blindness (if left untreated), Japanese patients with nAMD have been shown to frequently visit an ophthalmologist, which results in a significant burden on patients and impacts their ability to work [4].

Vascular endothelial growth factor-A (VEGF-A) plays a key role in nAMD [5] and anti-VEGF therapy, which requires regular intravitreal injections [6], has become the current standard-of-care treatment for nAMD in Japan [7]. However, observational studies show that patients with nAMD receive fewer anti-VEGF injections in clinical practice than in clinical trials, which may contribute to the worse visual outcomes seen in clinical practice [8–10]. This undertreatment of nAMD in clinical practice may be related to the burden of frequent monitoring and intravitreal injections with anti-VEGF treatment [11–13]. Therefore, therapeutic options that can ease symptoms and treatment burden on patients are required.

Abnormal angiogenesis contributes to various ocular disease, including nAMD. The angiopoietin-1 (Ang-1)–Tie signalling pathway is a regulator of angiogenesis. Angiopoietin-2 (Ang-2) is an agent that destabilises vessels by blocking Ang-1/Tie2 activation, with this destabilisation sensitising vessels to VEGF. In nAMD, Ang-1 signalling is inhibited by upregulated Ang-2, which synergises with VEGF-A to cause vascular hyperpermeability, neovascularisation, and inflammation [14]. Faricimab is the first humanised, bispecific, monoclonal immunoglobulin G antibody designed for intravitreal injection that independently binds and neutralises both Ang-2 and VEGF-A [15]. The pivotal phase III TENAYA and LUCERNE trials compared faricimab 6.0 mg (up to Q16W) with aflibercept 2.0 mg (Q8W) in patients with nAMD [16]. Results from these trials demonstrated that BCVA change from baseline to 1 year (averaged over weeks 40, 44, and 48) with faricimab dosed up to Q16W was non-inferior to aflibercept Q8W. Furthermore, anatomical outcomes and rates of ocular adverse events were similar between faricimab and aflibercept up to week 48. These findings demonstrated the efficacy and safety of faricimab in patients with nAMD.

The phenotype of Japanese patients with nAMD differs from White patients, with Japanese patients demonstrating a higher frequency of polypoidal choroidal vasculopathy (PCV), a lower frequency of bilateral presentation, and a greater predominance in men [17]. Thus, evaluating the effects of faricimab in Japanese patients with nAMD is of clinical relevance. To this point, consistent with the global findings, year 1 findings from the TENAYA Japan subgroup demonstrated that faricimab had vision and anatomical benefits similar to those of aflibercept, with extended durability and an acceptable safety profile [18].

In year 2 of the TENAYA and LUCERNE trials, a treat-and-extend–based (T&E) personalised treatment interval (PTI) regimen was introduced to allow retreatment to be guided by the individual patient’s disease activity [16]. Global results from the TENAYA and LUCERNE trials showed that personalised faricimab dosing up to Q16W maintained vision gains and controlled anatomic outcomes through year 2 [19]. This manuscript summarises 2-year efficacy, durability, and safety results for the TENAYA Japan subgroup treated with faricimab. We also report pooled global data from the TENAYA/LUCERNE trials.

## Materials and methods

### Study design

The study design for TENAYA (and identically designed LUCERNE) has been previously described in full [16] as has the TENAYA Japan subgroup [18]. TENAYA (NCT03823287) and LUCERNE (NCT03823300) trials were conducted over 112 weeks across 271 clinical sites worldwide, including 41 TENAYA sites in Japan. Shortly after the conclusion of global enrolment, additional patients were enrolled in a Japan extension of TENAYA to ensure there was an appropriate number to support faricimab registration in Japan. Study protocols were approved by the appropriate regulatory authorities, applicable institutional review boards, and ethics committees, and were conducted in accordance with the Declaration of Helsinki and principles of Good Clinical Practice.

Eligible patients were aged 50 years or older at randomisation and had presence of treatment-naïve choroidal neovascularisation (CNV) secondary to nAMD; subfoveal CNV or juxtafoveal or extrafoveal CNV, with subfoveal component related to CNV activity, confirmed on fluorescein angiography, and CNV exudation confirmed on spectral-domain optical coherence tomography (SD-OCT); CNV lesion size of ≤ 9 disc areas and CNV component area ≥ 50% of total lesion area; and Early Treatment Diabetic Retinopathy Study BCVA 78−24 letters, inclusive (20/32–20/320 approximate Snellen equivalent). All participants provided written informed consent.

### Treatment protocol

Details on randomisation and masking have been described previously [16]. Briefly, patients were randomised to faricimab or aflibercept. Patients in the faricimab arm received intravitreal faricimab 6.0 mg Q4W up to week 12 (4 initial injections) and, following protocol-defined disease activity criteria assessments at weeks 20 and 24, received faricimab Q8W, Q12W, or Q16W up to week 60. From week 60, patients in the faricimab arm were treated according to a protocol-driven T&E-based PTI regimen (Table S1), where dosing intervals could be extended in 4-week increments or reduced in 4- or 8-week increments to a minimum of Q8W, a maximum of Q16W, or maintained based on pre-specified criteria of BCVA, SD-OCT–determined central subfield thickness (CST) measurements, and presence of new macular haemorrhage. Patients in the aflibercept arm received intravitreal aflibercept 2.0 mg Q4W up to week 8 (3 initial injections) followed by fixed Q8W dosing. All patients attended study visits every 4 weeks up to the final visit at week 112 and received study treatment or sham injections (to preserve masking at non-active dosing visits) up to week 108.

### Outcome measures

Outcomes for this analysis were assessed for the TENAYA Japan subgroup and are presented alongside the pooled global TENAYA/LUCERNE cohort [19]. Secondary efficacy end points reported herein are consistent with the pre-specified end points in the primary analysis [16], including change in BCVA from baseline at 2 years (averaged over weeks 104, 108, and 112) and over time; the change in SD-OCT–measured CST from baseline at 2 years (averaged over weeks 104, 108, and 112) and over time; the proportion of patients on Q16W, Q12W, and Q8W dosing at week 112; and the incidence and severity of ocular adverse events (AEs) in the study eye and non-ocular AEs through study end.

### Statistical analysis

As previously described for the year 1 analyses [18], the TENAYA Japan subgroup efficacy analyses included all patients randomised at TENAYA trial sites in Japan and in the TENAYA Japan extension cohort. The pooled global TENAYA and LUCERNE efficacy analyses included all patients randomised in the trials.

Safety analyses for the TENAYA Japan subgroup and for the pooled global TENAYA/LUCERNE cohort included all patients in the respective cohorts who received at least 1 injection of study treatment (faricimab or aflibercept) in the study eye, grouped according to actual treatment received. Safety was assessed through descriptive summaries of ocular and non-ocular AEs, deaths, and ocular assessments through study end. AEs were coded using the Medical Dictionary for Regulatory Activities, version 24.1.

Adjusted means for continuous end points were assessed using a mixed model for repeated measures. Weighted proportions of binary end points were estimated using the Cochran–Mantel–Haenszel method.

COVID-19–related intercurrent events were handled using a hypothetical strategy where all values were censored after the intercurrent event, and non–COVID-19–related intercurrent events were handled using a treatment policy where all observed values were used regardless of occurrence of the intercurrent event.

No formal statistical testing of the comparison between faricimab and the active comparator, aflibercept, was performed for the Japan subgroup.

Statistical analyses were performed using SAS 9.4 (SAS Institute, Inc., Cary, NC, USA).

## Results

### Patient disposition

As previously reported, a total of 1329 patients were enrolled in the phase III global TENAYA and LUCERNE trials [16]. A total of 133 Japanese patients were enrolled in TENAYA (faricimab, *n* = 66; aflibercept, *n* = 67); 52 during global enrolment and 81 during the Japan extension [18]. Most patients in the faricimab and aflibercept arms in the pooled global TENAYA/LUCERNE cohort [19] and the TENAYA Japan subgroup completed the study through week 112 (Fig. S1).

In the TENAYA Japan subgroup, 12 patients discontinued treatment (4 withdrawal by patient, 4 adverse event, 3 physician decision, and 1 lack of efficacy).

### Baseline characteristics

As previously reported, baseline characteristics were generally balanced between treatment arms in the TENAYA Japan subgroup [18].

### Vision outcomes

**Fig. 1:**
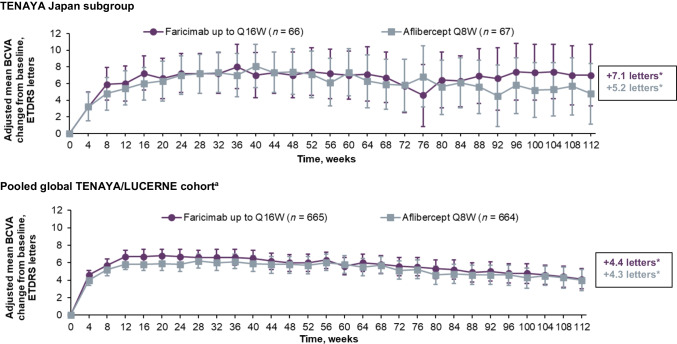
Adjusted mean change in BCVA from baseline through week 112 for the TENAYA Japan subgroup and the pooled global TENAYA/LUCERNE cohort. ^a^Pooled global cohort includes patients in Japan who were enrolled during the global phase of TENAYA/LUCERNE. Data originally published in Khanani et al. [19]. *Adjusted mean BCVA change from baseline averaged over weeks 104, 108, and 112. *BCVA* best-corrected visual acuity, *ETDRS* Early Treatment Diabetic Retinopathy Study, *Q8W* every 8 weeks, *Q16W* every 16 weeks

In the TENAYA Japan subgroup, vision gains from baseline achieved during year 1 were maintained during year 2, with comparable vision gains between treatment arms (Fig. 1). The adjusted mean (95% CI) BCVA change from baseline at year 2 (averaged over weeks 104, 108, and 112) was +7.1 (3.7–10.5) and +5.2 (1.9–8.6) letters in the faricimab and aflibercept arms, respectively; the difference in adjusted means was 1.9 letters (–2.9 to 6.7).

In the pooled global TENAYA/LUCERNE cohort, the adjusted mean (95% CI) BCVA change from baseline at year 2 (averaged over weeks 104, 108, and 112) was +4.4 (3.2–5.5) and +4.3 (3.1–5.4) letters in the faricimab and aflibercept arms, respectively (Fig. 1); the difference in adjusted means was 0.1 (–1.5 to 1.7) letters [19].

### Durability outcomes

**Fig. 2:**
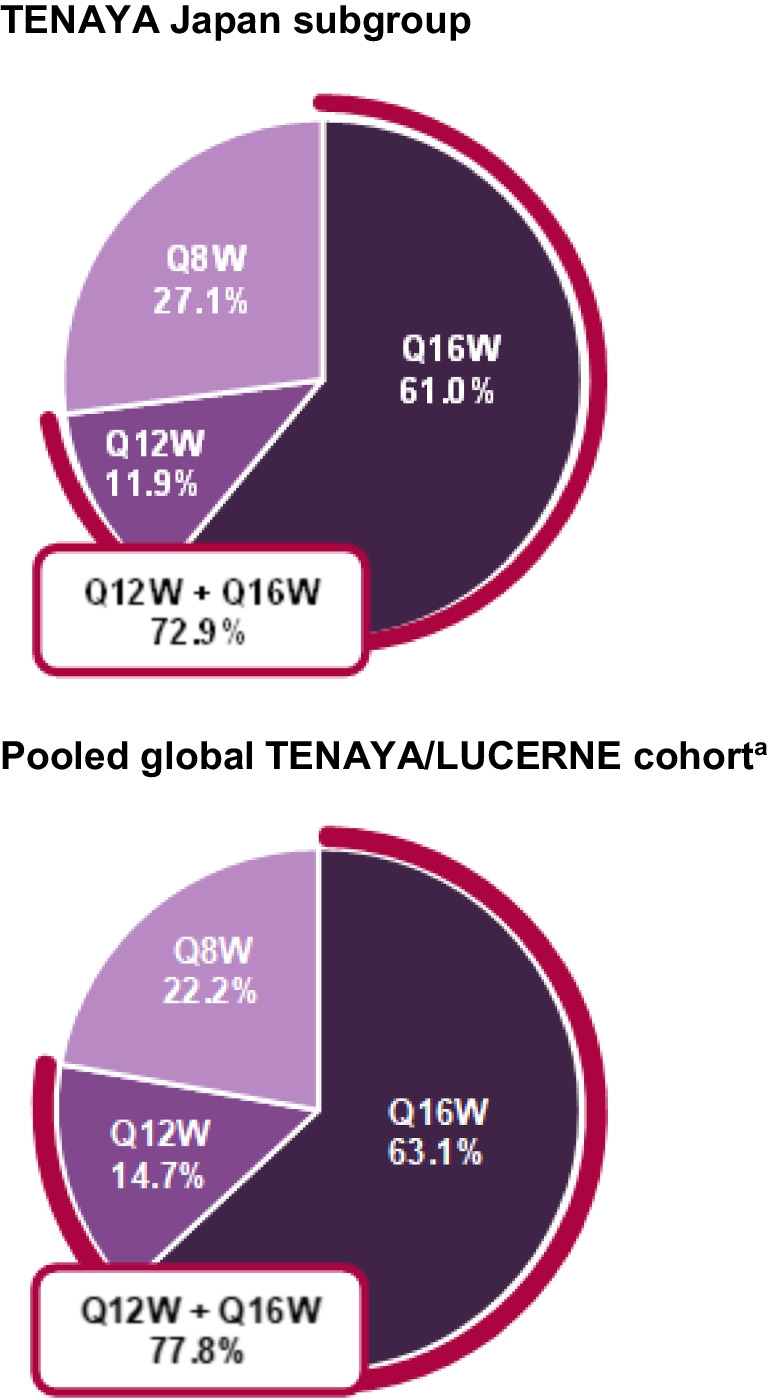
Proportion of patients in the faricimab up to Q16W arms who achieved Q8W, Q12W, or Q16W dosing at week 112 for the TENAYA Japan subgroup and the pooled global TENAYA/LUCERNE cohort. ^a^Pooled global cohort includes patients in Japan who were enrolled during the global phase of TENAYA/LUCERNE. Data originally published in Khanani et al. [19]. *Q8W* every 8 weeks, *Q12W* every 12 weeks, *Q16W* every 16 weeks

In the TENAYA Japan subgroup, the proportion of faricimab-treated patients on Q16W dosing was maintained from 66.1% at week 48 [18] to 61.0% at week 112, while the proportion on Q12W dosing decreased from 22.6% at week 48 to 11.9% at week 112. The proportion on extended (Q12W + Q16W) dosing remained high between week 48 (88.7%) [18] and week 112 (72.9%) (Fig. 2 and Fig. S2). The proportion of patients on Q8W dosing increased from 11.3% at week 48 to 27.1% at week 112. Some patients (4/7) on Q8W dosing at week 48 were able to extend to Q16W dosing by week 112. Over 2 years, the median number of study drug administrations in the TENAYA Japan subgroup was 10 injections in the faricimab arm and 15 injections in the aflibercept arm. During year 2, the median number of study drug administrations in the TENAYA Japan subgroup was 3 and 6 injections, respectively. Treatment interruptions during year 2 in the TENAYA Japan subgroup were experienced by 7 faricimab-treated patients and 5 aflibercept-treated patients.

In the pooled global TENAYA/LUCERNE cohort, the proportion of faricimab-treated patients on Q16W dosing increased from 45.3% at week 48 to 63.1% at week 112, and the proportion on extended (Q12W + Q16W) dosing remained similar between week 48 (78.8%) and week 112 (77.8%) (Fig. 2) [19]. Over 2 years, the median number of study drug administrations was 10 injections in the faricimab arm and 15 injections in the aflibercept arm [19]. During year 2, the median number of study drug administrations in the TENAYA/LUCERNE cohort was 3 and 6 injections, respectively [19]. Treatment interruptions during year 2 in the TENAYA/LUCERNE cohort were experienced by 43 faricimab-treated patients and 39 aflibercept-treated patients [19].

### Anatomic outcomes

**Fig. 3:**
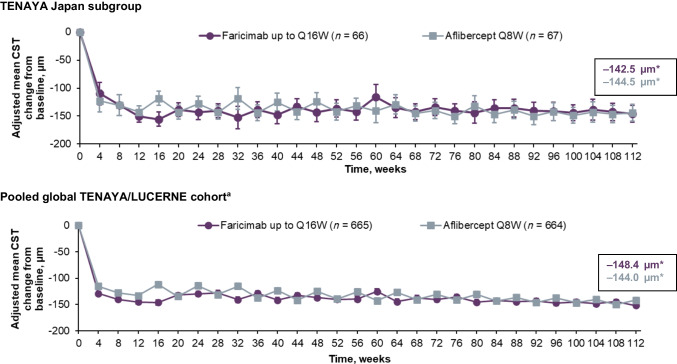
Adjusted mean change in CST from baseline through week 112 for the TENAYA Japan subgroup and the pooled global TENAYA/LUCERNE cohort. ^a^Pooled global cohort includes patients in Japan who were enrolled during the global phase of TENAYA/LUCERNE. Data originally published in Khanani et al. [19]. *Adjusted mean CST change from baseline averaged over weeks 104, 108, and 112. *CST* central subfield thickness, *Q8W* every 8 weeks, *Q16W* every 16 weeks

In the TENAYA Japan subgroup, CST decreases from baseline achieved during year 1 were maintained during year 2, with comparable decreases between treatment arms (Fig. 3). The adjusted mean (95% CI) CST decrease from baseline at year 2 (averaged over weeks 104, 108, and 112) was –142.5 μm (–157.4 to –127.6) and –144.5 μm (–159.1 to –129.9) in the faricimab and aflibercept arms, respectively; the difference in adjusted means was 2.1 μm (–18.8 to 23.0).

In the pooled global TENAYA/LUCERNE cohort, the adjusted mean (95% CI) CST decrease from baseline at year 2 (averaged over weeks 104, 108, and 112) was –148.4 μm (–152.7 to –144.2) and –144.0 μm (–148.3 to –139.8) in the faricimab and aflibercept arms, respectively (Fig. 3); the difference in adjusted means was –4.4 μm (–10.4 to 1.6) [19].

### Safety and tolerability

Consistent with the pooled global TENAYA/LUCERNE cohort [19], faricimab was well tolerated through week 112 in the TENAYA Japan subgroup (Table 1). The proportion of patients with ocular AEs in the study eye through week 112 was similar between treatment arms in the TENAYA Japan subgroup (faricimab, 31 [47.0%] patients; aflibercept, 30 [44.8%] patients) and the pooled global TENAYA/LUCERNE cohort (faricimab, 358 [53.9%] patients; aflibercept, 345 [52.1%] patients) [19]. The proportion of patients with serious ocular AEs in the study eye through week 112 was low and similar between treatment arms in the TENAYA Japan subgroup (faricimab, 6 [9.1%] patients; aflibercept, 6 [9.0%] patients) and the pooled global TENAYA/LUCERNE cohort (faricimab, 29 [4.4%] patients; aflibercept, 29 [4.4%] patients) [19]. No adverse events of retinal pigment epithelial tears were reported in the TENAYA Japan subgroup.

**Table 1:** Summary of key adverse events through week 112 for the TENAYA Japan subgroup and the pooled global TENAYA/LUCERNE cohort

	TENAYA Japan		Pooled global TENAYA/LUCERNE^a^	
	Faricimab up to Q16W (*n* = 66)	Aflibercept Q8W (*n* = 67)	Faricimab up to Q16W (*n* = 664)	Aflibercept Q8W (*n* = 662)
Total number of AEs^b^	320	248	3284	3321
Total number of SAEs^b^	24	20	280	380
Patients with any ocular AE, *n* (%)^c^	31 (47.0%)	30 (44.8%)	358 (53.9%)	345 (52.1%)
Patients with any ocular SAE, *n* (%)^c^	6 (9.1%)	6 (9.0%)	29 (4.4%)	29 (4.4%)
Patients with any treatment-related ocular AE, *n* (%)^c^	7 (10.6%)	6 (9.0%)	26 (3.9%)	19 (2.9%)
Patients with any treatment-related ocular SAE, *n* (%)^c^	1 (1.5%)	2 (3.0%)	10 (1.5%)	2 (0.3%)
Patients with any AEs of IOI, *n* (%)^c,d^	3 (4.5%)	0	20 (3.0%)	15 (2.3%)
Iritis	2 (3.0%)	0	8 (1.2%)	3 (0.5%)
Uveitis	1 (1.5%)	0	4 (0.6%)	3 (0.5%)
Keratic precipitates	0	0	2 (0.3%)	0
Vitritis	1 (1.5%)	0	4 (0.6%)	1 (0.2%)
Iridocyclitis	0	0	2 (0.3%)	1 (0.2%)
Chorioretinitis	0	0	1 (0.2%)	0
Post-procedural inflammation	0	0	0	5 (0.8%)
Non-infectious endophthalmitis	0	0	0	1 (0.2%)
Patients with ocular SAE known to be associated with anti-VEGF, *n* (%)				
Endophthalmitis	1 (1.5%)	0	3 (0.5%)	2 (0.3%)
Rhegmatogenous retinal detachment	1 (1.5%)	0	1 (0.2%)	0
Retinal tear	0	0	0	2 (0.3%)
Retinal pigment epithelial tear	0	0	4 (0.6%)	0
Intraocular pressure increased	0	0	1 (0.2%)	1 (0.2%)
Cataract traumatic	0	0	1 (0.2%)	1 (0.2%)
Patients with retinal occlusive vasculitis event, *n* (%)	1 (1.5%)^e^	0	0	0
Patients with retinal occlusive event, *n* (%)^f^	0	0	1 (0.2%)	0
Retinal vein occlusion	0	0	0	0
Retinal artery occlusion	0	0	0	0
Retinal artery embolism	0	0	1 (0.2%)^g^	0
Patients with any APTC events, *n* (%)^h^	0	0	22 (3.3%)	20 (3.0%)

*AE* adverse event, *APTC* Antiplatelet Trialists’ Collaboration, *IOI* intraocular inflammation, *Q8W* every 8 weeks, *Q16W* every 16 weeks, *SAE* serious adverse event, *VEGF* vascular endothelial growth factor

^a^Pooled global cohort includes patients in Japan who were enrolled during the global phase of TENAYA/LUCERNE. Data originally published in Khanani et al. [19]

^b^Total number of AEs and SAEs includes non-ocular and ocular events in the study or fellow eye

^c^AEs and SAEs in the study eye only are presented

^d^Excluding endophthalmitis

^e^When the Central Reading Centre (Reading Centre) reviewed the imaging tests performed at the onset of the adverse event, it was judged that there was no finding of retinal vasculitis obstructive

^f^Non-IOI related

^g^Hollenhorst plaque that was reported at the end of year 1 and was not treatment-related as per the investigator

^h^APTC events were adjudicated by an external independent committee; all other events were investigator reported

Percentages are based on *n* in the column headings. Multiple occurrences of the same AE in an individual are counted only once, except for the “Total number of AEs” and “Total number of SAEs” rows, in which multiple occurrences of the same AE are counted separately

The proportion of patients with intraocular inflammation (IOI) events in the study eye through week 112 was low in the TENAYA Japan subgroup (faricimab, 3 [4.5%] patients; aflibercept, 0 [0%] patients) and was similar between treatment arms in the pooled global TENAYA/LUCERNE cohort (faricimab, 20 [3.0%] patients; aflibercept, 15 (2.3%) patients) [19]. In the Japan subgroup, there was a low incidence of serious ocular AEs and IOI in both treatment arms. In the Japan extension study, there was 1 event of retinal occlusive vasculitis reported in the faricimab treatment arm. The event was reported as non-serious and resolved without treatment 1 month after onset. Visual acuity at the time of event onset was 66 letters, which improved to 79 letters upon event resolution. The diagnosis of retinal occlusive vasculitis could not be confirmed through an independent reading centre review of the ocular images taken at the time of the event.

In the Japan subgroup, there were 78.8% (52/66) non-ocular AEs reported in the faricimab arm and 79.1% (53/67) reported in the aflibercept arm.

There were no adjudicated Antiplatelet Trialists’ Collaboration events or deaths reported.

## Discussion

Previously reported 1-year TENAYA Japan subgroup analysis results [18] showed that faricimab up to Q16W had sustained efficacy, extended durability, and an acceptable safety profile, consistent with the global TENAYA/LUCERNE findings. The results presented herein show that the visual and anatomic improvements and extended durability seen with faricimab at year 1 in the TENAYA Japan subgroup were maintained through year 2, during which patients followed a T&E-based PTI regimen. Notably, the year 2 Japan subgroup findings were generally consistent with the pooled global TENAYA/LUCERNE results [19].

Year 1 BCVA gains with faricimab (+7.1 letters) [18] continued through year 2 (+7.1 letters) in the TENAYA Japan subgroup. The stability of BCVA gains with extended dosing may reflect faricimab’s Ang-2 inhibitory effects, promoting vascular stability. Interestingly, this contrasts with the pooled global TENAYA/LUCERNE cohort, where there was a trend for reduced BCVA gains over time despite robust and sustained CST reductions [19], which is a similar observation to that reported in other trials [20, 21]. The cause of this underlying drift in BCVA gains over time warrants further investigation.

The proportion of patients in the TENAYA Japan subgroup on extended dosing remained high at year 2 and comparable to that in the pooled global TENAYA/LUCERNE cohort (the proportion of patients on extended dosing increased between year 1 and 2 in the pooled global cohort [19]). A slightly lower proportion of patients in the TENAYA Japan subgroup were on faricimab Q16W dosing at year 2 compared with year 1 (61.0% vs 66.1%); however, fewer patients were on faricimab Q12W dosing (11.9% vs 22.6%) and more patients were on faricimab Q8W dosing (27.1% vs 11.3%). The proportion of patients on faricimab Q8W dosing at year 2 was slightly higher in the TENAYA Japan subgroup compared with the pooled global TENAYA/LUCERNE cohort (27.1% vs 22.2%) [19]. These findings may reflect PCV activity during year 2. Specifically, the PCV phenotype being more common in Japanese patients than in White patients may be a factor related to some patients requiring more frequent treatment during year 2. The proportion of patients with PCV was higher in the Japan subgroup than the pooled global TENAYA/LUCERNE cohort (31.3% vs 4.8%) [18]. Interestingly, 2 recent real-world studies in Japan demonstrated complete regression of polypoidal lesions following short-term treatment with faricimab [22, 23]. To further evaluate the possible impact of PCV and because there was a relatively low number of patients with PCV at baseline in the current analysis, the SALWEEN trial (ISRCTN69073386) will investigate the efficacy, durability, and safety of faricimab over 108 weeks in patients with PCV from China, Hong Kong, India, Japan, South Korea, Malaysia, Singapore, Taiwan, and Thailand. Of note, some patients on Q8W dosing at week 48 were able to extend to Q16W dosing by week 112, while some patients on Q12W dosing at week 48 were able to extend to Q16W dosing by week 112. This increase in the dosing interval over the long term may have been due to Ang-2–related vascular stabilisation.

Consistent with the BCVA findings and the global findings [19], CST reductions with faricimab at year 1 (–140.6 μm) [18] were also maintained through year 2 (–142.5 μm) in the TENAYA Japan subgroup. These CST reductions with faricimab were similar to those seen with aflibercept but with fewer injections.

Consistent with the global TENAYA/LUCERNE findings [19], faricimab continued to be well tolerated in the TENAYA Japan subgroup through year 2. As per the 1-year results [18], rates of ocular AEs and ocular inflammation were low. Further information on longer-term safety (and indeed efficacy) of faricimab will come from the AVONELLE-X extension trial (NCT04777201) for patients completing TENAYA/LUCERNE and the VOYAGER study (NCT05476926) involving patients with nAMD or diabetic macular oedema who are treated with faricimab or the Port Delivery System with ranibizumab in clinical practice.

This analysis of the TENAYA Japan subgroup through year 2 has some limitations. The trial was not designed to evaluate the head-to-head durability of faricimab with aflibercept. Specifically, aflibercept was administered Q8W without the possibility to extend treatment intervals. Despite the enrolment of additional patients in a Japan extension of TENAYA, the sample size was small. Polypoidal choroidal vasculopathy was not fully diagnosed in this study as indocyanine green angiography imaging was optional; therefore, efficacy in these patients could not be confirmed.

In conclusion, visual and anatomic improvements, extended durability, and an acceptable safety profile were maintained through 2 years with faricimab up to Q16W in the TENAYA Japan subgroup. The Japan subgroup findings continued to be generally comparable to those observed in pooled global TENAYA/LUCERNE cohort [19]. The proportion of patients on faricimab extended dosing at week 48 (88.7%) remained high at week 112 (72.9%). These data provide further support for dual Ang-2/VEGF-A inhibition with faricimab as a novel treatment that can provide durable efficacy, extended treatment intervals, and, therefore, a decreased treatment burden for patients with nAMD.

### Supplementary Information

Below is the link to the electronic supplementary material.

Supplementary file1 (PDF 207 KB)

Supplementary file2 (PDF 578 KB)

Supplementary file3 (PDF 736 KB)

## Data Availability

For eligible studies, qualified researchers may request access to individual patient-level clinical data through a data request platform. At the time of writing this request platform is Vivli. https://vivli.org/ourmember/roche/. For up-to-date details on Roche’s Global Policy on the Sharing of Clinical Information and how to request access to related clinical study documents, see here: https://go.roche.com/data_sharing. Anonymised records for individual patients across more than 1 data source external to Roche cannot, and should not, be linked due to a potential increase in risk of patient re-identification.
