# Supplementary material for: Efficacy, durability, and safety of faricimab up to every 16 weeks in patients with neovascular age-related macular degeneration: 2-year results from the Japan subgroup of the phase III TENAYA trial
Source: Graefes Arch Clin Exp Ophthalmol. 2024 Mar 14;262(8):2439–48. doi: 10.1007/s00417-024-06377-1 (PMC11271316; doi:10.1007/s00417-024-06377-1)
Supplement: Supplementary file 1 — Supplementary file1 (PDF 207 KB) [file 417_2024_6377_MOESM1_ESM.pdf]

Table S1. Treat-and-extend−based personalised treatment interval algorithm conclusions

Dosing Interval	Criteria
**Interval extended by 4 weeks (to a maximum of Q16W)**	• Stable CST[a] compared with the average of the last two study drug dosing visits, and no increase ≥ 50 μm in CST *(*compared with the lowest on-study drug dosing visit measurement*)*<br>**AND**<br>• No decrease ≥ 5 letters in BCVA[b] compared with the average from the last two study drug dosing visits, and no decrease ≥ 10 letters in BCVA[b] compared with the highest on-study drug dosing visit measurement<br>**AND**<br>• No new macular haemorrhage[c]
**Interval reduced (to a minimum Q8W**)<br>If one of the criteria was met, the interval was reduced by 4 weeks.<br>If two or more criteria were met or one criterion included new macular haemorrhage, the interval was reduced to an 8-week interval[d]	• Increase ≥ 50 μm in CST compared with the average from the last two study drug dosing visits or ≥ 75 μm compared with the lowest on-study drug dosing visit measurement<br>**OR**<br>• Decrease ≥ 5 letters in BCVA[b] compared with average of last two study drug dosing visits or decrease ≥ 10 letters in BCVA[b] compared with the highest on-study drug dosing visit measurement<br>**OR**<br>• New macular haemorrhage[c]
**Interval maintained**	• If extension or reduction criteria were not met

[a]Where stability was defined as a change of CST of > 30 μm.

[b]Change in BCVA should be attributable to nAMD disease activity (as determined by investigator).

[c]Refers to macular haemorrhage owing to nAMD activity (as determined by investigator).

[d]Patients whose treatment interval was reduced by 8 weeks from Q16W to Q8W were not allowed to return to a Q16W interval during the study.

BCVA, best-corrected visual acuity; CST, central subfield thickness; nAMD, neovascular age-related macular degeneration; Q8W, every 8 weeks; Q16W, every 16 weeks.
