# Supplementary material for: Efficacy, durability, and safety of faricimab up to every 16 weeks in patients with neovascular age-related macular degeneration: 2-year results from the Japan subgroup of the phase III TENAYA trial
Source: Graefes Arch Clin Exp Ophthalmol. 2024 Mar 14;262(8):2439–48. doi: 10.1007/s00417-024-06377-1 (PMC11271316; doi:10.1007/s00417-024-06377-1)
Supplement: Supplementary file 2 — Supplementary file2 (PDF 578 KB) [file 417_2024_6377_MOESM2_ESM.pdf]

Fig. S1. Patient flow diagram for the TENAYA Japan subgroup at week 112.

Q8W, every 8 weeks; Q16W, every 16 weeks.

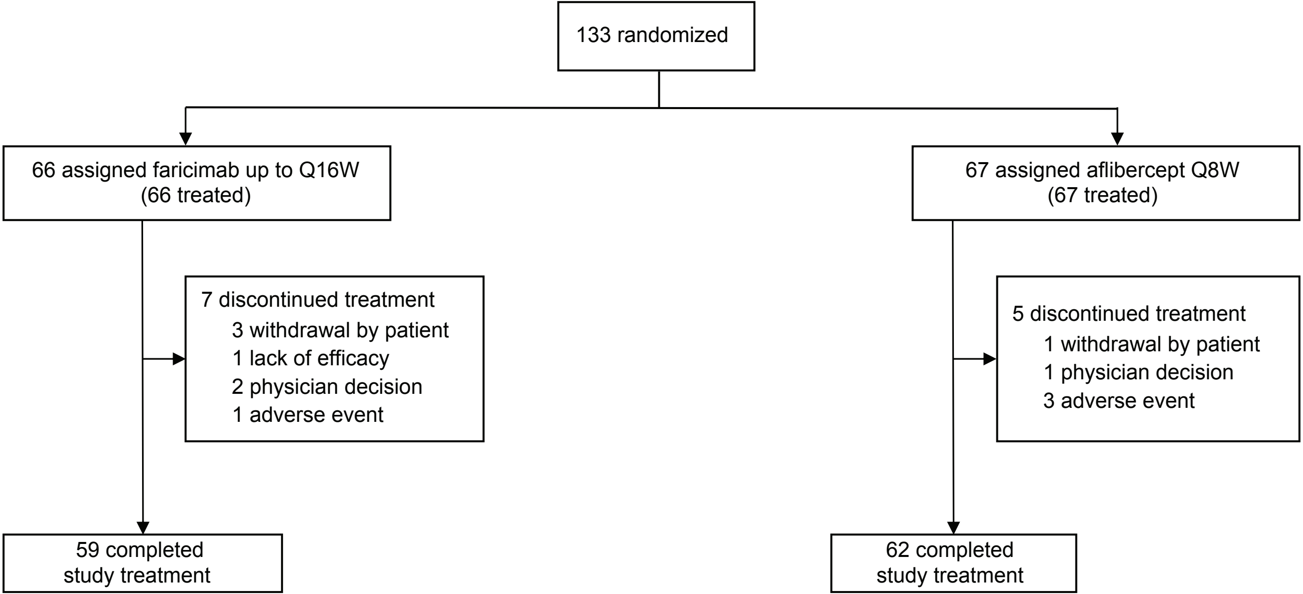
133 randomized
66 assigned faricimab up to Q16W
(66 treated)
67 assigned aflibercept Q8W
(67 treated)
7 discontinued treatment
3 withdrawal by patient
1 lack of efficacy
2 physician decision
1 adverse event
5 discontinued treatment
1 withdrawal by patient
1 physician decision
3 adverse event
59 completed
study treatment
62 completed
study treatment
