# Supplementary material for: Efficacy, durability, and safety of faricimab up to every 16 weeks in patients with neovascular age-related macular degeneration: 2-year results from the Japan subgroup of the phase III TENAYA trial
Source: Graefes Arch Clin Exp Ophthalmol. 2024 Mar 14;262(8):2439–48. doi: 10.1007/s00417-024-06377-1 (PMC11271316; doi:10.1007/s00417-024-06377-1)
Supplement: Supplementary file 3 — Supplementary file3 (PDF 736 KB) [file 417_2024_6377_MOESM3_ESM.pdf]

Fig. S2. Heatmap showing faricimab treatment interval from day 1 through week 112 in the TENAYA Japan subgroup.

Q4W, every 4 weeks; Q8W, every 8 weeks; Q12W, every 12 weeks; Q16W, every 16 weeks; T&E, treat-and-extend.

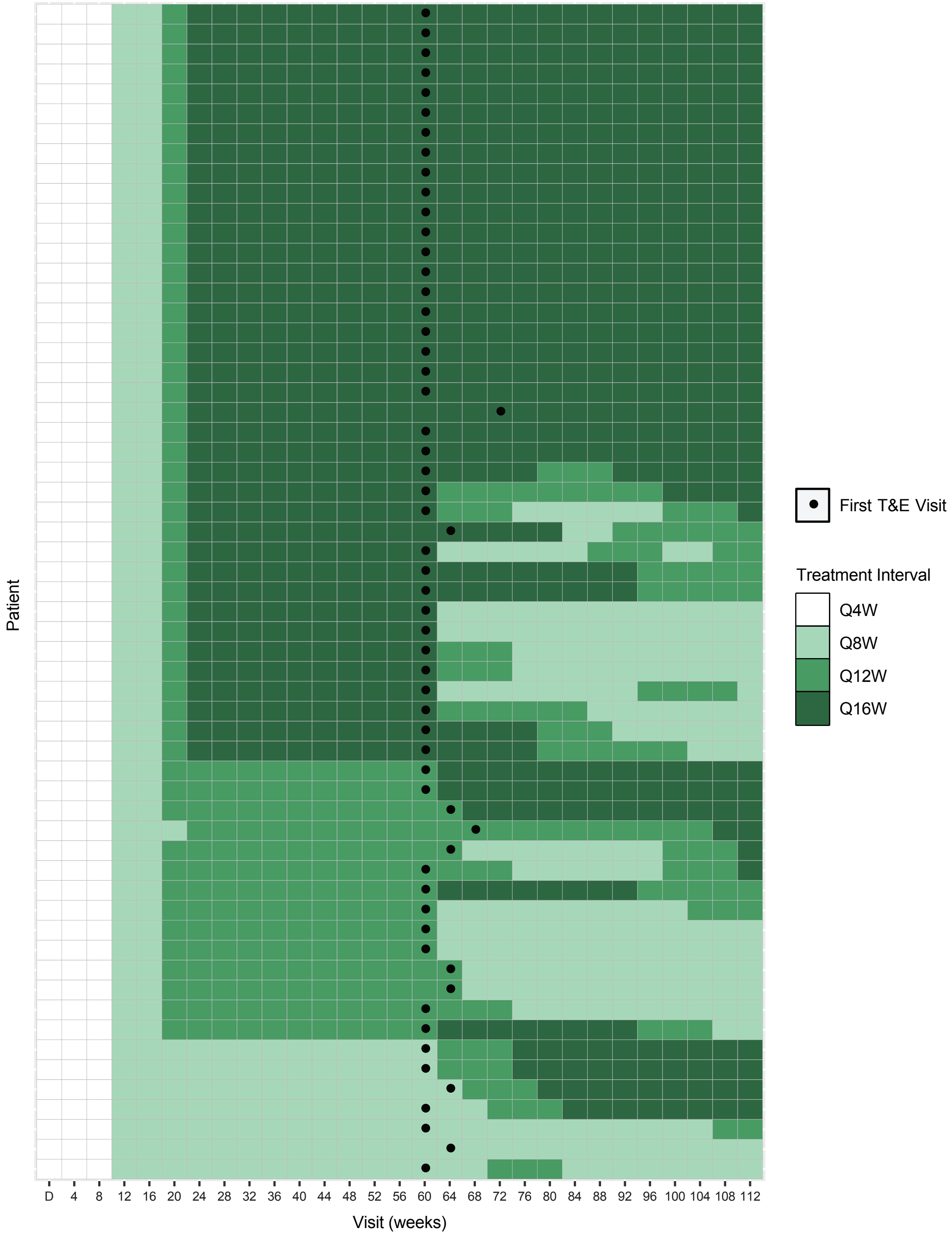

Patient
D
4
8
12
16
20
24
28
32
36
40
44
48
52
56
60
64
68
72
76
80
84
88
92
96
100
104
108
112
Visit (weeks)
First T&E Visit
Treatment Interval
Q4W
Q8W
Q12W
Q16W
